# Death patterns among Nigerian leaders

**DOI:** 10.5249/jivr.v2i2.30

**Published:** 2010-06

**Authors:** Kenneth C Eze, Ozoemenam M Ugochukwu, Martin A Nzegwu

**Affiliations:** ^*a*^Department of Radiology, Faculty of Medicine, Nnamdi Azikiwe University Teaching Hospital, Nnewi, Anambra State, Nigeria; ^*b*^Department of History and International Studies, University of Benin, Benin City, Nigeria; ^*c*^Department of Morbid Anatomy, University of Nigeria Teaching Hospital Enugu, Nigeria

## Abstract

**Background:**

The aim of this study is to establish the patterns of death amongst Nigerian leaders since independence, thus providing a feasible avenue to avoid their recurrence if possible especially amongst the political elite who currently hold power.

**Methods:**

Using available unclassified authentic public information, all leaders who had ruled Nigeria since her independence on 1 October, 1960 until her 45th birthday on 1 October 2005, irrespective of whether they are dead or alive were included. Data was extracted and analyzed.

**Results:**

On 1 October 2005, Nigeria celebrated 45 years as a sovereign nation. Within this period, the country has had eleven leaders, all of whom were men. Only three (27.3%) were civilians, while eight (72.7%) were army generals. Of the eleven leaders, four (36.4%) had died before Nigeria reached its 45th birthday and all of these four (100%) died while still in office. Three of the dead leaders (75%) were assassinated, while one (25%) died suddenly in mysterious circumstances, believed to be the result of poisoning by unknown external powerful interest groups. Three of the deaths (75%) occurred during violent periods of Nigeria's checkered history (1966-1970 and 1993-1999), showing that periods of national and international strife appeared to be the weakest link in chains of events that led to their death while in office. Autopsies were neither requested nor performed on any of the dead leaders, signifying an entrenched culture of nonchalance, a lack of a coordinated national coroner's law and contempt for accurate and detailed death records. Worse still, no valid tenable death certificate has ever been issued. In other words, no attempt has been made to determine the cause of death of four of the nation's former leaders. Only hurried national burials were accorded two (50%) of them while the other two (50%), who died in the coup and revenge coup of 1966, were completely neglected, and not even given a decent national burial.

**Conclusions:**

The facts identified above will serve as a landmark to highlight an existing problem, and thus bring the issue to the attention of policy-makers and the political elite. The overall expected benefit is that nations like Nigeria can focus on the issue of orderly succession and the peaceful handing-over of government to duly transparently elected national leaders and all efforts should be made to avoid holding on to power unnecessarily. The tenets of democracy shall be upheld and transparent elections take place so as to reduce national tension and strife to the barest minimum. We also strongly recommend a review and improvement of Nigeria's national coroner's laws.

## Introduction

Since Nigeria’s independence from Great Britain on 1 October1960, several leaders or heads of governments have died, most of them while in office, and no organized or formal study has attempted to document the pattern of death amongst these leaders, despite the fact that unclassified credible information is available in the public domain, including newspaper reports, history books, world almanacs and social studies papers.

 In 1996, the World Health Assembly declared violence as a leading global public health problem.^1^  The paper presented then also cited reports by some authors,^1,2^ which showed that world leaders most commonly died by violence, usually assassinations, with 44% dying violently while in office and 11% dying violently out of office. An unacceptably high level of violent deaths of world leaders has occurred world-wide, but most frequently in developing countries and especially the Middle-East, South-East Asia and sub-Saharan Africa.^2,3^ Some of these developing countries, especially in sub-Saharan Africa, are also known to have very small volumes of available data for recording and assessing the immediate and remote causes of such deaths among their leaders, as well as for other health indices.^2,4^

Other reports on the pattern of death among leaders of developed countries found stroke, heart disease and cancer to be the major natural causes of death.^5,6^ These reports also indicate the decreasing trend of coronary heart disease and stroke and find that these causes of death are even less common in leaders than in the general population. Even when they occurred, they seldom led to disruption of power unlike in developing countries.^6^ In developing countries, while infectious diseases are the leading cause of death in the general population, they rank extremely low as a cause of death amongst leaders. Among young men in sub-Saharan Africa and most other developing countries, homicide, war, road traffic injury and infectious diseases are the leading causes of death.^1,3,7,8^ In women of childbearing age, hemorrhage, infection, hypertension and unsafe abortion are major causes of death.^9,10^ It is therefore clear that the leading causes of death among the general population in developing countries are not the same as those afflicting the leaders of such countries.^2,5,6^

This study examines the patterns of death in Nigerian leaders since independence, in order to bring them to the attention of the national political elite and policy-makers. Thus, in agreement with Shiffman,^11^ other political scientists and public policy researchers, identifying the contributing factors of these deaths, which would otherwise have remained hidden to policy-makers, will elicit the required change faster. The deeper significance of statistics is that they are not only useful for monitoring purpose but can also act as a catalyst for action.^12^

## Methods

The information for this study was obtained over a period of 18 months from public domains including Nigerian national websites, world almanacs, books of Who‘s Who in Nigeria and African nation and Nigerian year books.^13-17^ The relevant information extracted from these published authenticated sources, include the names of Nigerian heads of state, their age, how long they were in office, factors that led to their emergence as leaders as well as the circumstances surrounding their exit. For those who died, data was available regarding the date of death, the cause of death and political factors associated with their emergence and exits from office. As an exclusion criteria, only information collaborated by all sources was included while any information not agreed upon by the sources and incompatible with events at the time was excluded. Limited publications in this field as well as the exclusion criteria are responsible for the relatively few references used for the study.

## Results

Nigeria gained it independence from Great Britain on 1 October 1960 and celebrated 45 years as a sovereign nation in December 2005. Within this period, she had eleven (11) leaders, all of whole were men. Only three (27.3%) were civilians while eight (72.7%) were military generals. One of the military leaders (Olusegun Obasanjo) was returned as an elected civilian president, many years after serving and retiring as a military head of state.

Four of the leaders had died before the end of the period, three (75%) having been assassinated, while one (25%) died suddenly in mysterious circumstances, believed by most Nigerians to have been the result of poisoning by as-yet unnamed powerful external interest groups following the country’s instability after annulment by a military head of state of the democratic elections, held on 12 June 1993.^13-15^ All four (100%) dead leaders died while still in office. The oldest of these four leaders when taking up office was General Sani Abacha, who was 50 years old and the youngest was General Murtala Mohammed, who came to power at the age of 38. The longest stay in office among the dead leaders was 6 years by Tafawa Balawa, followed by 5 years by General Sani Abacha. The remaining two were assassinated within 8 months of assuming office. Those who are still alive are much older than the average life expectancy of the average Nigerian male (51 years).^13-14^

Three (75%) of the four dead leaders were from the northern part of Nigeria. Two leaders (50%) died in 1966 in events that eventually lead to a civil war, while one (25%) died in 1976 and the last one (25%) in 1998. See Table 1.

All deaths were sudden and apparently unnatural, emphasizing the speed with which the lethal events caused their deaths. Autopsies were neither requested nor performed on any of them. Consequently no valid tenable death certificate has ever been issued in these cases and coroner’s inquests have never been held to ascertain the valid cause of death of four of the nation’s former leaders. Hurried national burials were arranged for two of the leaders (50%) while the other two (50%), who died in the coup and revenge coup of 1966, didn’t even receive a decent national burial.

## Discussion

The existence of only a small amount of adequate and credible literature on the subject matter, possibly due to fear of retribution following the repression associated with decades of military rule, as well as the need to corroborate fully the available information sources before use, may constitute some limitation to the study. Violent death by assassination was the mode of death of three (75%) of the four dead leaders. This percentage is more than the average world-wide figure for violent death of national leaders reported to be 44% while in office and 11% after leaving office.^2^ The percentage of violent causes of death among leaders in Nigeria (75%) is almost double the reported figure for the rest of the world (44%). These two figures are also comparatively higher than the percentage of deaths by violence among the world’s population at large, reported at 3.5%.^3^ The fact that no Nigerian head of state died of natural causes further highlights the major significance of violence, especially assassination, in the epidemiology of deaths among Nigerian leaders. One leader (Sani Abacha) died in circumstances which many Nigerians believe may have been masterminded by unnamed external powerful interest groups.^13-15^This fact is especially corroborated by the public testimony of his trusted aid (Al Mustapha), who publicly acknowledged that he died (by asphyxiation) in similar circumstances to those surrounding the death of MKO Abiola, the opposition leader widely thought to have won the democratic elections of 12 June 1993. These two deaths were thought to be the cause of the national crisis that threatened Nigeria’s existence following the annulment of the aforesaid elections. Sani Abacha died eating apple, while Abiola was said to have died after drinking tea. Furthermore, both deaths occurred in the presence of foreign visitors.^15^ Stroke and coronary heart disease are major causes of death among leaders from other parts of the world outside office,^2,5,6^ but in Nigeria were the deaths of leaders are mainly associated with violent causes. It is also worth noting that all deaths of these Nigerian leaders occurred while they were still in office.

**Table:** Table 1: **Nigerian leaders and cause of their death**

no	Name	Name	State of Origin	Born	Died /Alive	Aged at 1st October 2005 (Years)	AAO (Years)	DO (Years)	Mode of entry	Time in office	Cause of death	SHO	OSS
1	H1	Balewa	Bauchi	12/1912	15/1/1966	54	48	5.29	Civil	1/10/1960 to 15/1/1966	A	No	Yes
2	H2	Ironsi	Abia	3/3/1924	29/7/1966	42	42	0.54	Force *	16/1/1966 to 29/7/1966	A	No	_
3	H3	Gowon	Plateau	19/10/1934	Alive	71	33	9	Force	1/8/1966 to 29/7/1975	_	No	Yes
4	H4	Murtala	Kano	8/11/1938	13/2/1976	37	37	.54	Force	29/7/1975 to 13/2/1976	A	_	_
5	H5	Obasanjo	Ogun	5/3/1937	Alive	68	39	3.63	Force **	13/2/1976 to 1/10/1979	A	Yes	No
6	H6	Shagari	Sokoto	25/5/1925	Alive	80	54	4.25	Civil	1/10/1979 to 31/12/1983	_	No	Yes
7	H7	Buhari	Katsina	17/12/1942	Alive	63	41	1.67	Force	1/1/1984 to 27/8/1985	_	_	_
8	H8	Babangida	Niger	17/8/1941	Alive	64	44	8	Force	27/8/1985 to 26/8/1993	_	No	Yes
9	H9	Shonekan	Ogun	9/5/1936	Alive	69	57	0.23	Extra-legal	26/8/1993 to 17/11/1993	_	_	No
10	H10	Abacha	Kano	20/9/1943	8/6/1998	54	50	4.55	Force	17/11/1993 to 8/6/1998	Natural#	No	Yes
11	H11	Abdusalami	Niger	14/6/1942	Alive	63	56	0.97	Force***	9/6/1998 to 29/5/1999	_	Yes	No
12	H5 $	Obasanjo$	Ogun	5/3/1937	Alive	68	61	6.33	Civil	29/5/1999 to 1/10/2005+	_		

* = Inherited office after unsuccessful coup d’état in January 1966 in which he did not participate, **=Inherited office after an unsuccessful coup d’état in which he did not participate led to the assassination of his immediate boss, the head of state. ***= A military junta selected him after the sudden death of the head of state. #= Sudden unexplained death of the head of state believed in some quarters to been masterminded by external powers. + = In office at the time of this study. A = Assassination. AAO= Age at assumption of office, DO = Duration in office, SHO=Successful handover of office, OSS=Overstayed first term/self-allotted time. $= Second term of H5.

Since 75% of the dead leaders were army generals in military government, only 12 out of Nigeria’s 45 years of independence (26.7%) were periods ruled by civilians while the other 33 years (73.3) was under military rule. Currently all living Nigerian leaders are aged above the average life expectancy of Nigerians, and their life expectancy thus far also compares favorably with those of other Nigerians of the same socio-economic status. As previously stated, the deaths of three (75%) out of the four leaders who died in office occurred in violent periods of Nigeria’s checkered history (1966-1970 and 1993-1999) showing that periods of national and international strife appear to be the weakest link in chains of events that lead to the death of leaders while still in office.^1,2,3,4,8^ These weak links are generally characterized by a series of civil revolts, riots, national strikes, clamp-downs on the free press and major human rights abuses, sparking major foreign opposition and the imposition of economic sanctions on Nigeria. It must be noted that it was at periods such as these that the most deaths occurred.

Three (75%) of the four dead Nigerian leaders were of northern extraction while one (25%) was from the south east, alluding to the fact that out of the past 11 leaders, including those alive, 8 (72.7%) were from northern Nigeria, and only 3 (27.3%) were from southern Nigeria. Furthermore, during the 45 years of Nigerian independence, northern Nigerians have ruled for 33 (73.3%) while leaders from the South have ruled for 12 (26.7%) years with very poor attempts to deepen democratic values and sustain democracy based on free, fair and transparent elections, ensuring a free press and promoting the rule of law, equality and social justice. This study will therefore recommend that everyone must work to focus on the issue of orderly succession and the peaceful handing-over of government to duly transparently elected national leaders. All efforts should be made to avoid holding on to power unnecessarily and to promote the tenets of democracy and transparent election.

In conclusion, the paper observes that the percentage of violent deaths among Nigerian leaders, which stands at 75%, far exceeds that found amongst other world leaders (44%), and posits that a lack of a transparent and credible democratic order, which ensures orderly ascent and transition to power and good governance, may be the single most important reason for these avoidable deaths. The paper strongly recommends a reappraisal of national Nigerian coroner’s law with a view to improving them.
